# Spanish translation, cultural adaptation and validation of the SarQoL®: a specific health-related quality of life questionnaire for sarcopenia

**DOI:** 10.1186/s12891-022-05125-y

**Published:** 2022-03-01

**Authors:** Beatriz Montero-Errasquín, Nieves Vaquero-Pinto, Vicente Sánchez-Cadenas, Anton Geerinck, Elisabet Sánchez-García, Jesús Mateos-Nozal, José Manuel Ribera-Casado, Alfonso J. Cruz-Jentoft

**Affiliations:** 1 Servicio de Geriatría, Hospital Universitario Ramón Y Cajal, IRYCIS Madrid, Ctra Colmenar km 9,100, 28034 Madrid, Spain; 2 Division of Public Health, Epidemiology and Health Economics, University of Liège, Place du 20 Août 7, 4000 Liège, Belgium; 3 Facultad de Medicina, Universidad Complutense de Madrid, Pl. de Ramón y Cajal, s/n, 28040 Madrid, Spain

**Keywords:** Health surveys, Muscle function, Physical functional performance

## Abstract

**Background:**

In 2015, a specific health-related quality of life questionnaire for sarcopenia, SarQoL®, was developed and validated in French. Since then, SarQoL® has been adapted and validated in different languages. We prepared a translation, cultural adaptation and validation of the psychometric properties of the SarQoL® into Spanish.

**Methods:**

A cross-sectional study with 86 participants. The translation and adaptation followed international guidelines with two direct translations, a synthesized version of the direct translations, two reverse translations, consensus by an expert committee of a pre-final version, pre-test by end users and final version. The discriminative power (logistic regression analyses), construct validity (Pearson and Spearman´s correlation), internal consistency (Cronbach´s alpha coefficient), test–retest reliability (intraclass correlation coefficient) and ceiling and floor effects were analyzed.

**Results:**

The Spanish version showed good construct validity (high correlation with comparable domains of the SF-36), high internal consistency (Cronbach's alpha coefficient: 0.84) and excellent test–retest reliability (ICC: 0.967, 95%, CI 0.917 – 0.989). However, it had no discriminative power between sarcopenic and non-sarcopenic participants defined with the EWGSOP and FNIH diagnostic criteria of sarcopenia. It did show discriminative power between patients with decreased vs normal muscle strength (54.9 vs. 62.6, p 0.009) and low vs. normal physical performance (57.3 vs. 70.2; p 0.005). No ceiling or floor effect was found.

**Conclusions:**

The Spanish version of SarQoL® has similar psychometric properties to those of the original version of the instrument. It did not discriminate between sarcopenic and non-sarcopenic patients diagnosed according to the EWGSOP or FNIH criteria, but it did with those with low muscle strength and low physical performance.

**Supplementary Information:**

The online version contains supplementary material available at 10.1186/s12891-022-05125-y.

## Background

Sarcopenia is a progressive and generalized disease of skeletal muscle with accelerated loss of muscle mass and function [1]. Sarcopenia is prevalent in the older population, associated with multiple related factors such as aging, different diseases, treatments, living and environmental conditions, and with adverse outcomes such as functional decline, increased risk of falls, frailty, fractures, increased health costs and mortality [1–8]. Therefore, sarcopenia and its adverse consequences may be associated with a worse quality of life [9, 10].

Health-related quality of life is measured in clinical practice using questionnaires that address different domains. Such instruments are part of the wider concept of Patient Reported Outcome Measures (PROM) and are gaining momentum as relevant information to be collected in diseases and interventions. Quality of life questionnaires and instruments are usually self-administered, can be generic or specific for a given condition and usually gauge the impact of a disease on different domains of the quality of life of the patients that may be impaired by that condition [11, 12].

Interest in quality of life in sarcopenic patients is growing. Some studies have used general instruments for its assessment, such as SF-36 or EQ-5D [9, 13, 14]. However, data on quality of life in sarcopenia are heterogeneous, because different diagnostic criteria are used to define sarcopenia [15] and generic quality of life questionnaires may not address the specific impact of this condition on quality of life [10, 16]. For this reason, Beaudart et al. developed and validated in Belgium in 2015 a specific instrument—in French language—to measure the quality of life in sarcopenia, named SarQoL® (www.sarqol.org) [17, 18]. It is a self-administered questionnaire, which takes approximately 10–15 min to complete, with 22 questions on 55 aspects of quality of life, organized around 7 domains: physical and mental health, mobility, body composition, functionality, activities of daily living, leisure activities and fears. The questions are evaluated according to a Likert scale. It scores on a scale from 0 to 100, a higher score means a better quality of life. This instrument has been validated in English [19, Additional file 1], Romanian [20,] Dutch [21], Polish [22], Russian [23], Lithuanian [24], Greek [25] Chinese [26] and Turkish [27]. A Spanish psychometric validation of SarQoL, not the full validation, was published recently [28] using our Spanish translation that was available on the SarQoL website after presentation at a congress while we conducted the validation study, but it used a less reliable methodology so their validation has some limitations.

Spanish is the native language of more than 500 million persons, so our aim was to translate, adapt and validate the psychometric properties of the SarQoL® in Spanish language using the best available methodology for questionnaire validation.

## Methods

### Study population

Patients were screened from those who volunteered to participate in a European multicenter study on physical exercise and nutritional intervention to improve physical performance in patients with frailty and sarcopenia (*SPRINT-T*) [29]. Inclusion criteria for our study were: age 65 years or older, a *Short Physical Performance Battery (*SPPB) [30] score ≤ 9, who had Spanish as their mother tongue and who completed and signed the informed consent form disregarding if they met or not inclusion criteria for *SPRINT-T*. Participants with cognitive impairment were excluded. Main sociodemographic variables (age, gender, civil status and academic level) were self-reported. Medical conditions, drugs and functional status (Barthel Index and FAC) were established through self-reported history and medical records. Measurements of anthropometric variables and SPPB were performed by the study staff.

### Assessment of sarcopenia

Sarcopenia was defined according to two different diagnostic criteria:

- the original *European Working Group on Sarcopenia in Older People* (EWGSOP) definition in 2010 [31]. Based on the suggested cut-off points, we chose the following:

- low muscle mass, with cut-off points of < 7.26 kg/m2 for men and < 5.5 kg/m2 for women, measured with a dual energy x-ray absorptiometry (DXA).
- low muscle strength (< 30 kg for men and < 20 kg for women) measured with a manual hydraulic dynamometer Jamar model according to the Southampton protocol [32].
- low physical performance, measured with a ≤ 9 score on the Short Physical Performance Battery (SPPB).

- the *Foundation for the National Institutes of Health* (FNIH) criteria [33, 34]:

- low muscle mass adjusted by the body mass index: appendicular lean mass/body mass index (ALM/BMI) < 0.789 for men and < 0.512 for women, measured with a dual energy x-ray absorptiometry (DXA).
- low muscle strength (< 26 kg for men and < 16 kg for women) measured with a manual hydraulic dynamometer Jamar model according to the Southampton protocol.

With evolving changes in sarcopenia definitions, we decided also to classify our participants with low physical performance (SPPB < 8) and low muscle strength according to the original definition of the EWGSOP (31) (low handgrip strength: < 30 kg for men and < 20 kg for women) as per protocol and also with its last update, the EWGSOP2 (3) (low handgrip strength: < 27 kg for men and < 16 kg for women). 

### Spanish translation and adaptation of the SarQoL®

The process of translation and adaptation of the original questionnaire into Spanish was performed following the five phases recommended in international guides for intercultural adaptation of self-administered scales [35–40]:

- *Direct translations from French to Spanish:* two translations were made from the original French version into Spanish by two native Spanish translators who are bilingual for French.
- *Summary of the two direct translations:* the two previous translators made a synthesis of their two direct translations to achieve a single tentative Spanish version of SarQoL®.
- *Backward translations from Spanish version into French:* two different native French translators, bilingual for Spanish and blind to the original version of SarQoL®, translated the Spanish version back into French. This reverse translation ensures that the Spanish version accurately reflects the contents and meaning of the original French version.
- *Review by an expert committee:* a group of experts consisting of a medical professional, two methodologists, a Spanish academic teacher and the four translators compared the original French version with all the translations and agreed on a pre-final Spanish version of SarQoL®.
- *First evaluation of the Spanish version:* the pre-final Spanish version was completed by 10 participants to ensure they understood each question of the questionnaire, and minor changes were performed to obtain the final Spanish version used in the validation study. The time needed to complete the questionnaire was also measured.

### Validation of psychometric properties

At present, there is no consensus on specific recommendations for the validation of a translated questionnaire [41], but most general recommendations used in the literature propose the following steps along validation process [35, 36, 39, 42, 43], which were used in the original questionnaire and, therefore, were followed to validate the Spanish version.

#### Sample size

The appropriate sample size for validation and proposed by the authors of the original questionnaire is based on Terwee's recommendations: a sample of 100 participants with at least 50 in the target population that the instrument is intended to measure (persons with sarcopenia) [44].

#### Discriminative power

The hypothesis is that the quality of life is better in participants without sarcopenia than in sarcopenic ones. Total score of the SarQol® questionnaire and individual domains scores from two groups were compared using logistic regression analyses adjusted for clinical characteristics which were significantly different between groups in univariate analysis.

#### Internal consistency

This is an estimation of the homogeneity and the degree of coherence across all the items of the scale. Internal consistency reliability was determined using Cronbach's alpha coefficient. A value greater than 0.70 indicates a good level of internal consistency. The impact of each domain was also evaluated. The correlation of each domain with the total score was analyzed using correlations analysis. A correlation greater than 0.81 was considered excellent, between 0.61 and 0.80 very good, between 0.41 and 0.60 good, between 0.21 and 0.40 acceptable and below 0.20 insufficient.

#### Construct validity

It measures correspondence between the observed variables and the theoretical construct to be measured, reflecting whether the questionnaire measures what it intends to measure and how it relates to other questionnaires or tests that measure the same domains. In addition to SarQoL®, sarcopenic participants completed two general quality of life scales: the Short Form-36 (SF-36) [45] and the Euro-QoL 5 domains (EQ-5D) [46, 47]. The copyright holders of the Short Form-36 (SF-36) and the Euro-QoL 5 domains (EQ-5D) authorized the use. Construct validity was measured by convergent and divergent validity. Pearson and Spearman correlations were used to assess the correlation between similar domains in SarQoL® and the other two questionnaires for convergent validity (physical function, limitation caused by physical problems, pain, general health status, vitality in SF-36 and mobility and usual activities in EQ-5D). Spearman's correlations were used to compare the different domains of these two questionnaires with the SarQoL® global score (social function, limitation caused by affective problems and mental health in the SF-36 and self-care, pain/discomfort and anxiety/depression in the EQ-5D).

#### Test–retest reliability

It refers to the degree of coincidence of the test results when the questionnaire is completed at different times over time under the same vital circumstances. For this purpose, participants completed the SarQoL® for a second time two weeks after filling it for the first time. The intraclass correlation coefficient (ICC) was used to determine the reliability of the global score and each domain between the two questionnaires. An ICC greater than 0.7 is considered acceptable.

#### Ceiling and floor effect

It shows when a high percentage of participants have the highest and lowest score in the scale. These groups should not exceed 15% to be considered non-significant [18].

### Statistical analysis

*IBM SPSS Statistics* software version 24.0.0 was used. The distribution of quantitative variables was tested with the Shapiro–Wilk test. Quantitative variables with a normal distribution were expressed as mean ± SD, quantitative variables who showed a non-normal distribution were expressed with interquartile range (IQR) and nominal variables were reported as absolute and relative frequencies (%). Differences of characteristics between sarcopenic and non-sarcopenic participants were tested with the parametric Student’s T test or the non-parametric Mann–Whitney U test for quantitative variables and with a Chi-squared test or a Fisher exact test for nominal variables. Results were considered statistically significant at the 5% critical level (*P* < 0.05).

## Results

### Participants

The baseline characteristics of all participants (*n* = 86) are described in Table 1. The median age was 77 years (range 70–91 years), 80.2% women. Depending on the different diagnostic criteria and sarcopenia cut-off points used, the prevalence of sarcopenia in the sample varied. Thus, the prevalence was 18.6% with the EWGSOP criteria and 15.1% with the FNIH criteria. The prevalence of participants with low handgrip strength according to the EWGSOP and EWGSOP2 criteria was 58.1%, and 30.2% respectively. The prevalence of participants with low physical performance was 73.2%.

**Table 1:** Baseline characteristics of participants

Age [years: mean ± SD (interval)]	77.6 ± 5.3 (70 – 91)
Female sex [n (%)]	69 (80.2)
Academic level [n (%)]:	
No studies	4 (4.7)
Primary	27 (31.4)
Secondary	41 (47.7)
Universitary	14 (16.3)
Civil status [n (%)]:	
Single	7 (8.1)
Married	31 (36.0)
Divorced	4 (4.7)
Widower	44 (51.2)
Living situation [n (%)]:	
Alone	38 (44.2)
Partner	33 (38.4)
Family	14 (16.3)
Nursing home	1 (1.2)
Comorbidities [nº: mean ± SD (interval)]	4.7 ± 1.9 (0 – 9)
Drugs [nº: mean ± SD (interval)]	6.5 ± 3.3 (0 – 16)
Barthel index (mean ± SD; interval)	96.4 ± 3.2 (85 – 100)
FAC 5 [n (%)]	85 (98.8)
SPPB (mean ± SD; interval)	6.8 ± 1.5 (3 – 9)
Hand grip strength (mean ± SD; interval)	19.9 ± 8.1 (2 – 45)
Woman	17.2 ± 5.2 (2 – 29)
Man	31.4 ± 7.5 (13 – 45)
Weight [kg: mean ± SD (interval)]	69.9 ± 13.1 (43.8 – 109.5)
Woman	67.5 ± 11.0 (43.8 – 105.6)
Man	79.7 ± 16.7 (55.0 – 109.5)
Height [m: mean ± SD (interval)]	1.5 ± 0.1 (1.4 – 1.7)
Woman	1.5 ± 0.1 (1.4 – 1.7)
Man	1.6 ± 0.1 (1.5 – 1.7)
BMI [kg/m^2^: mean ± SD (interval)]	28.9 ± 5.0 (18.6 – 48.8)
Woman	28.9 ± 4.9 (18.6 – 48,8)
Man	29.2 ± 5.3 (20.0 – 41.2)
ALM [kg: mean ± SD (interval)]	16.1 ± 3.6 (11.4 – 30.8)
Woman	14.8 ± 1.9 (11.4 – 20.9)
Man	21.3 ± 4.3 (14.9 – 30.8)
ALM/BMI (mean ± SD; interval)	0.5 ± 0.1 (0.4 – 0.9)
Woman	0.5 ± 0.6 (0.4 – 0.8)
Man	0.7 ± 0.1 (0.6 – 0.9)
Arm circumference [cm (mean ± SD; interval)]	29.7 ± 3.8 (19.0 – 39.4)
Woman	29.8 ± 3.8 (19.0 – 39.4)
Man	29.1 ± 3.9 (22.0 – 34.7)
Waist circumference [cm (mean ± SD; interval)]	97.6 ± 12.1 (69.5 – 132.5)
Woman	95.6 ± 11.0 (69.5 – 132.5)
Man	105.4 ± 13.7 (82.5 – 126.2)
Calf circumference [cm (mean ± SD; interval)]	35.2 ± 3.5 (28.0 – 47.9)
Woman	35.0 ± 3.4 (28.0 – 47.9)
Man	36.1 ± 3.9 (30.0 – 45.0)

Notes: *SD* standard desviation, *FAC* Functional Ambulation Categories, *SPPB* Short Physical Performance Battery, *nº* number, *BMI* Body Mass Index, *ALM* appendicular lean mass

### Translation

The Spanish version of SarQoL® was translated following international recommendations without relevant issues. Ten participants completed the pre-test version in 15–20 min. Most of them reported some problems in understanding the concept of muscle mass and the multiple-box format questionnaire. The final version is shown in Additional file 2.

### Psychometric properties

#### Discriminative power

Table 2 shows the total and individual domain scores of the SarQoL® questionnaire for non-sarcopenic and sarcopenic participants defined by EWGSOP and FNIH criteria. The SarQoL questionnaire showed similar results in sarcopenic and non-sarcopenic participants. In fact, there are domains with higher (better) scores in sarcopenic compared to nonsarcopenic participants. Therefore, we could not confirm the discriminative power of this questionnaire with these diagnostic criteria.

**Table 2:** The SarQoL® scores according diagnostic criteria

EWGSOP CRITERIA			
	NON SARCOPENIC (*n*= 70)	SARCOPENIC (*n*=16)	p*
SarQoL D1	58.87 (52.20 – 68.87)	70.54 (59.70 – 78.87)	0.029
SarQoL D2	59.72 (46.53 – 75.00)	69.44 (53.47 – 76.39)	0.224
SarQoL D3	62.50 (50.00 – 70.83)	68.75 (52.08 – 78.13)	0.271
SarQoL D4	62.02 (51.34 – 70.02)	74.04 (64.56 – 82.69)	0.006
SarQoL D5	48.33 (40.00 – 63.75)	62.92 (50.83 – 74.58)	0.009
SarQoL D6	33.25 (33.25 – 66.50)	49.88 (33.25 – 66.50)	0.214
SarQoL D7	87.50 (75.00 – 87.50)	87.50 (87.50 – 87.50)	0.198
Overall SarQoL	57.33 (49.41 – 66.45)	72.25 (59.07 – 77.89)	0.008
FNIH CRITERIA			
	NON SARCOPENIC (*n*= 73)	SARCOPENIC (*n*=13)	p*
SarQoL D1	62.20 (52.20 – 72.20)	58.87 (55.53 – 66.08)	0.880
SarQoL D2	61.11 (48.61 – 76.39)	61.11 (44.45 – 69.44)	0.299
SarQoL D3	62.50 (50.00 – 70.83)	70.83 (58.33 – 72.92)	0.430
SarQoL D4	65.38 (52.89 – 76.79)	53.85 (51.92 – 67.59)	0.216
SarQoL D5	51.67 (40.84 – 66.67)	51.67 (36.66 – 63.75)	0.458
SarQoL D6	33.25 (33.25 – 66.50)	49.88 (33.25 – 74.81)	0.387
SarQoL D7	87.50 (75.00 – 87.50)	75.00 (75.00 – 87.50)	0.061
Overall SarQoL	59.70 (51.48 – 73.75)	57.03 (50.59 – 65.38)	0.323

Notes: *EWGSOP* European Working Group on Sarcopenia in Older People, *FNIH* Foundation for the National Institutes of Health, D1 (mental and physical health), D2 (mobility), D3 (body composition), D4 (functionality), D5 (activities of daily living), D6 (leisure activities); D7 (fears)

*P-value obtained from linear regression with SarQoL scores as dependent variable and n° of comorbidities and sarcopenia status as independent variables

In contrast, when we classified participants according to strength and physical performance, the SarQol questionnaire did show discrimination power (Table 3).

**Table 3:** The SarQoL® scores according handgrip strength and physical performance

**EWGSOP (HANDGRIP STRENGTH < 30 KG/ < 20 KG**)			
	NORMAL HANDGRIP STRENGTH (*n* = 36)	LOW HANDGRIP STRENGTH (*n* = 50)	p*
SarQoL D1	69.42 (56.09 – 78.87)	58.32 (50.81 – 65.53)	0.001
SarQoL D2	69.44 (56.25 – 80.56)	58.33 (44.44 – 69.44)	0.003
SarQoL D3	66.67 (51.04 – 75.00)	62.50 (50.00 – 70.83)	0.420
SarQoL D4	70.39 (60.10 – 82.55)	59.27 (50.00 – 67.31)	0.002
SarQoL D5	56.67 (46.67 – 71.25)	50.00 (38.33 – 65.00)	0.092
SarQoL D6	49.88 (33.25 – 66.50)	33.25 (33.25 – 66.50)	0.290
SarQoL D7	87.50 (75.00 – 87.50)	87.50 (75.00 – 87.50)	0.050
Overall SarQoL	65.18 (55.43 – 76.93)	55.55 (49.14 – 63.40)	0.002
**EWGSOP2 (HANDGRIP STRENGTH < 27 KG/ < 16 KG)**			
	NORMAL HANDGRIP STRENGTH (*n* = 60)	LOW HANDGRIP STRENGTH (*n* = 26)	p*
SarQoL D1	63.31 (54.43 – 75.53)	56.64 (49.69 – 65.80)	0.030
SarQoL D2	66.67 (50.00 – 77.78)	52.78 (43.74 – 64.58)	0.010
SarQoL D3	64.58 (50.00 – 73.95)	64.58 (52.09– 70.83)	0.694
SarQoL D4	65.72 (56.25 – 80.36)	53.85 (50.96 – 67.31)	0.012
SarQoL D5	55.12 (45.35 – 71.25)	47.50 (38.33 – 60.00)	0.034
SarQoL D6	41.56 (33.25 – 66.50)	33.25 (33.25 – 66.50)	0.915
SarQoL D7	87.50 (75.00 – 87.50)	75.00 (75.00 – 87.50)	0.014
Overall SarQoL	62.61 (53.11 – 74.69)	54.92 (47.54 – 60.06)	0.009
**PHYSICAL PERFORMANCE (SPPB)**			
	NORMAL (*n* = 23)	LOW (*n* = 63)	p*
SarQoL D1	68.87 (55.53 – 78.87)	58.87 (52.20 – 68.87)	0.057
SarQoL D2	69.44 (52.78 – 77.78)	61.11 (44.44 – 72.22)	0.103
SarQoL D3	70.83 (50.00 – 79.17)	62.50 (50.00 – 70.83)	0.132
SarQoL D4	71.43 (55.77 – 80.77)	62.50 (50.00 – 69.64)	0.038
SarQoL D5	65.00 (51.67 – 73.33)	48.33 (38.33 – 60.00)	0.001
SarQoL D6	66.50 (33.25 – 66.50)	33.25 (33.25 – 66.50)	0.022
SarQoL D7	87.50 (75.00 – 87.50)	87.50 (75.00 – 87.50)	0.114
Overall SarQoL	70.23 (55.09 – 78.40)	57.29 (49.35 – 66.25)	0.005

Notes: *EWGSOP* European Working Group on Sarcopenia in Older People, *SPPB* Short Physical Performance Battery, D1 (mental and physical health), D2 (mobility), D3 (body composition), D4 (functionality), D5 (activities of daily living), D6 (leisure activities); D7 (fears)

^*^P-value obtained from linear regression with SarQoL scores as dependent variable and n° of comorbidities and sarcopenia status as independent variables

#### Internal consistency

The internal consistency is described in Table 4. Cronbach's alpha coefficient was 0.84, which is a very good level of internal consistency. The correlation of each domain with the total SarQol score was excellent (> 0.81) in domains D1 (mental and physical health), D2 (mobility), D4 (functionality) and D5 (activities of daily living), very good (> 0.61) in domain D3 (body composition) and good (> 0.41) in domains D6 (leisure activities) and D7 (fears).

**Table 4:** Internal consistency (Cronbach´s alpha coefficient)

Overall SarQol	0.836
D1	0.843
D2	0.856
D3	0.697
D4	0.913
D5	0.858
D6	0.442
D7	0.485

Notes: D1 (mental and physical health), D2 (mobility), D3 (body composition), D4 (functionality), D5 (activities of daily living), D6 (leisure activities); D7 (fears)

#### Construct validity

The SarQoL® total score showed a good correlation with similar domains of SF-36 such as physical function, limitation caused by physical problems, vitality and general health status with the EWGSOP diagnostic criteria, but not with pain. It also showed good correlation in similar domains of SF-36 such as physical function, limitation caused by physical problems and vitality with the FNIH criteria but not with pain and general health status. A good correlation was also found with similar EQ-5D domains of mobility and usual activities with the two diagnostic criteria used (Table 5). No significant correlations were found between SarQoL® and SF-36® or EQ-5D® for divergent correlation when the FNIH diagnostic criteria were used. Some significant correlations were found with some domains of the SF-36® such as the limitation caused by affective problems (correlation 0.683, *p* = 0.004) and mental health (correlation 0.648, *p* = 0.007) with the EWGSOP criteria. Overall, this confirms a good construct validity of the questionnaire.

**Table 5:** Construct validity

**EWGSOP CRITERIA (*n* = 16)**		
	Correlation	p*
SF – 36		
Physical function	0,652	0,006
Limitation caused by physical problems	0,569	0,021
Vitality	0,754	0,001
General health status	0,667	0,005
Pain	0,282	0,290
EQ – 5D		
Mobility	-0,624	0,010
Usual activities	-0,809	0,000
**FNIH CRITERIA (*n* = 13)**		
SF – 36		
Physical function	0,887	0,000
Limitation caused by physical problems	0,656	0,015
Vitality	0,651	0,016
General health status	0,212	0,486
Pain	0,216	0,478
EQ – 5D		
Mobility	-0,750	0,003
Usual activities	-0,615	0,025

Notes: *SF-36* Short Form-36®, *EQ – 5D* Euro-QoL® 5 domains

^*^p-value obtained from Pearson and Spearman correlations

#### Test–retest reliability

There was an excellent degree of agreement between the test and the retest completed 2 weeks later (Table 6). The intraclass correlation coefficient (ICC) was 0.967 (CI 0.917—0.989). The overall score and each domain present an ICC above 0.7 (except the D3 domain on body composition with the FNIH criteria) so that the Spanish version of the SarQoL is considered reliable.

**Table 6:** Test–retest reliability in sarcopenic participants

**EWGSOP CRITERIA (*n* = 16)**		
	ICC	CI 95%
SarQoL D1	0.931	0.816 – 0.975
SarQoL D2	0.893	0.541 – 0.968
SarQoL D3	0.883	0.703 – 0.957
SarQoL D4	0.929	0.811 – 0.975
SarQoL D5	0.964	0.901 – 0.987
SarQoL D6	0.885	0.703 – 0.958
SarQoL D7	0.789	0.457 – 0.923
Overall SarQoL	0.967	0.917 – 0.989
**FNIH CRITERIA (*n* = 13)**		
	ICC	CI 95%
SarQoL D1	0.733	0.352 – 0.909
SarQoL D2	0.863	0.612 – 0.956
SarQoL D3	0.697	0.288 – 0.895
SarQoL D4	0.855	0.595 – 0.953
SarQoL D5	0.924	0.768 – 0.976
SarQoL D6	0.987	0.958 – 0.996
SarQoL D7	0.742	0.334 – 0.914
Overall SarQoL	0.973	0.918 – 0.922

Notes: *EWGSOP* European Working Group on Sarcopenia in Older People, *FNIH* Foundation for the National Institutes of Health, D1 (mental and physical health), D2 (mobility), D3 (body composition), D4 (functionality), D5 (activities of daily living), D6 (leisure activities); D7 (fears)

#### Ceiling and floor effect

No sarcopenic or non-sarcopenic participants obtained the lowest score (0 points) or the highest score (100 points) when completing the Spanish version of SarQoL. Therefore, no ceiling or floor effect was found.

## Discussion

SarQoL® is the first specific health-related quality of life questionnaire developed for sarcopenia. This study was designed to create and validate a Spanish version of SarQoL® to be used in daily clinical practice and research in Spanish-speaking countries.

The first step was to complete the rigorous process of adapted translation following all the international recommendations. This process of translation and cultural adaptation was also followed in other translated versions of SarQoL®. The method of bilingual native translators and direct and reverse translations ensured objectivity and equivalence with the original French questionnaire, as confirmed by our good internal consistency and excellent test–retest reliability. These findings are in agreement with those described in previous validations [19–21].

The previously published Spanish validation [28] did not present the whole process from the beginning. In fact, they used our translated and adapted version of SarQoL® and demonstrated discriminative power but they assessed muscle mass with a less reliable method (bioelectrical impedance) with Asian cut-off points for a Spanish population.

Our Spanish version of SarQoL® found no difference in quality of life between sarcopenic and non-sarcopenic with the traditional definitions of sarcopenia, so its discriminative power could not be demonstrated and this was unexpected. In the validation studies of other translated versions, some discriminative power was observed, although the total score of the questionnaire and the partial scores of the different domains were greatly variable in each version, ranging from 50,3 in Lithuanian to 67,1 in Dutch in sarcopenic participants [21, 24]. This large variability in the scores in the different versions of the questionnaire could reflect the heterogeneity of the perception of quality of life in different countries. There has also been heterogeneity in the sample size and the diagnostic criteria and methods used to define sarcopenia. However, our Spanish questionnaire did show good discrimination according to muscle function (muscle strength or physical performance). This suggests that quality of life, at least in our participants, is better correlated to muscle function than to muscle mass and emphasized the issues raised with measuring muscle mass and defining cut-off points [2, 48]. Our cut-off point of SPPB ≤ 9 as an inclusion criterion is based on the design of the SPRINTT trial (based on the LIFE trial) [28, 49] and SPPB < 8 as an indicator of low physical performance and sarcopenia severity according to the EWGSOP2 when we classified our participants according to their physical function. The relation between quality of life and muscle function and not with sarcopenia was also described by Marques [50]. The updated definition of the EWGSOP2 [3] tries to overcome this problem by stating that a person with low muscle strength has probable sarcopenia, and in such patients, the Spanish version of SarQoL has in fact shown to be able to accurately measure quality of life. In fact, a short version of SarQoL has recently been published, also focused on low muscle strength, demonstrating excellent discrimination power comparing probable sarcopenia versus no sarcopenia according to EWGSOP2 criteria [51].

The rest of the psychometric properties of the Spanish version are maintained with respect to the original version. Our version of SarQoL® had an excellent internal consistency (Cronbach's alpha coefficient of 0.84) similar to that of the original questionnaire (Cronbach's alpha coefficient of 0.87). It also showed a significant correlation with similar quality of life domains of other two general quality of life questionnaires such as physical function, limitation caused by physical problems, vitality, mobility and usual activities that confirms the validity of the construct. The test–retest reliability of the Spanish version is excellent (CCI of 0.97), again close to that of the original SarQoL® (CCI of 0.91). In both versions, a ceiling and floor effect was not observed.

This study has some limitations. The sample size did not reach the target number of sarcopenic patients, which could have modified the analysis. For this reason, the sample was classified according to the grip strength and physical performance to achieve a larger group of participants with low muscle function. Therefore, our sample was not fully enriched with sarcopenic patients as defined by the initial criteria for sarcopenia, but did show reduced muscle function, a concept where the most current definitions of sarcopenia are focusing. However, in other validation studies of the scale (such as the original, English or Romanian) the target number of 50 sarcopenic was not reached either, and they did obtain discriminative power. Concerning the number of sarcopenic participants in the sample, the recently published short version of the original SarQoL has also been validated with a low number of participants with confirmed sarcopenia [51]. The scale validation sample sizes are generally similar to ours, so it is unlikely that increasing the sample size would change the results. Another limitation may be sample selection. Our participants were recruited from a European multicenter clinical trial that aims to demonstrate that protocolized physical exercise and nutritional intervention improves physical performance in sarcopenia patients. Some of our participants belonged to the intervention group and others had been excluded for different reasons, and this could have influenced their perception of quality of life. We chose these candidates because all participants had a DXA made by a well-trained technician, so we had reliable and homogeneous data on all of them for muscle mass.

This study also has strengths. First, we were in contact and collaboration with the authors of the original questionnaire from the beginning. In addition, we followed the strictest recommendations of international guidelines for the translation, cultural adaptation and validation of health-related quality of life scales.

## Conclusions

This study summarizes all the work done to translate, adapt, and validate the Spanish version of a specific health-related quality of life scale for sarcopenia, SarQoL®. Finding no differences in quality of life in sarcopenic participants with "traditional" definitions of sarcopenia, we focused on muscle function, and the Spanish version showed discriminative power. Muscle function seems to have a higher impact on quality of life than muscle mass.

## Supplementary Information

**Additional file 1.**

**Additional file 2.**

## Data Availability

The datasets used and/or analyzed during the current study are available from the corresponding author on reasonable request.

## Abbreviations

ALM: Appendicular Lean Mass.
BMI: Body Mass Index
DXA: Dual X-ray Absorciometry
EQ-5D: Euro-QoL® 5 domains
EWGSOP: European Working Group on Sarcopenia in Older People
FAC: Functional Ambulatory Category
FNIH: Foundation for the National Institutes of Health
ICC: Intraclass Correlation Coefficient
IQR: Interquartile Range
PROM: Patient Reported Outcome Measures
SarQoL: SARcopenia and Quality of Life
SF-36: Short Form-36®
SPPB: Short Physical Performance Battery
SPRINT-T: Sarcopenia and Physical fRailty IN older People: multi-componenT Treatment strategies
