# Supplementary material for: Spanish translation, cultural adaptation and validation of the SarQoL®: a specific health-related quality of life questionnaire for sarcopenia
Source: BMC Musculoskelet Disord. 2022 Mar 1;23:191. doi: 10.1186/s12891-022-05125-y (PMC8887022; doi:10.1186/s12891-022-05125-y)
Supplement: Supplementary file 2 — Additional file 2. [file 12891_2022_5125_MOESM2_ESM.pdf]

SarQoL
Sarcopenia and Quality of Life

Calidad de vida en la sarcopenia

Este cuestionario trata sobre la sarcopenia

Consiste en una debilidad muscular que aparece con la edad y que puede repercutir en su vida cotidiana. Esta encuesta nos permitirá saber si este estado muscular afecta a su calidad de vida actual.

Por favor, marque la respuesta más apropiada para cada pregunta. Responder a este cuestionario sólo le llevará unos 10 minutos.

1. Actualmente, ¿sufre usted una disminución:

	Mucho	Bastante	Un poco	Nada en absoluto
De fuerza en los brazos?	☐	☐	☐	☐
De fuerza en las piernas?	☐	☐	☐	☐
De masa muscular ?	☐	☐	☐	☐
De energía?	☐	☐	☐	☐
De capacidades físicas?	☐	☐	☐	☐
De flexibilidad muscular?	☐	☐	☐	☐

2. ¿Sufre usted dolores musculares?

- ☐ Frecuentemente
- ☐ De vez en cuando
- ☐ Casi nunca
- ☐ Nunca

Copyright© - January 2015 - All right Reserved

3. Cuando realiza esfuerzos físicos ligeros (andar despacio, planchar, limpiar el polvo, lavar los platos, hacer bricolaje, recoger piñas o frutos en el jardín, regar el jardín, etc.) ¿se resiente:

	Frecuentemente	De vez en cuando	Casi nunca	Nunca	Nunca realizo esfuerzos físicos de este tipo
De la dificultad?	☐	☐	☐	☐	☐
Del cansancio?	☐	☐	☐	☐	☐
Del dolor?	☐	☐	☐	☐	☐

4. Cuando realiza esfuerzos físicos moderados (andar rápido, limpiar los cristales, pasar el aspirador, lavar el coche, arrancar las malas hierbas del jardín, etc.) ¿se resiente:

	Frecuentemente	De vez en cuando	Casi nunca	Nunca	Nunca realizo esfuerzos físicos de este tipo
De la dificultad?	☐	☐	☐	☐	☐
Del cansancio?	☐	☐	☐	☐	☐
Del dolor?	☐	☐	☐	☐	☐

5. Cuando realiza esfuerzos físicos importantes (correr, hacer una excursión, levantar objetos pesados, mudarse, cavar en el jardín, etc.), ¿se resiente:

	Frecuentemente	De vez en cuando	Casi nunca	Nunca	Nunca realizo esfuerzos físicos de este tipo
De la dificultad?	☐	☐	☐	☐	☐
Del cansancio?	☐	☐	☐	☐	☐
Del dolor?	☐	☐	☐	☐	☐

6. Actualmente, ¿tiene usted el sentimiento de ser mayor?

- ☐ Sí, totalmente
- ☐ Sí, bastante
- ☐ Sí, un poco
- ☐ No, en absoluto

Copyright© - January 2015 - All right Reserved

7. Si la respuesta es sí, ¿qué es lo que le produce esta impresión?

(Puede elegir varias respuestas)

- ☐ Caigo enfermo más fácilmente
- ☐ Tomo muchos medicamentos
- ☐ Siento debilidad muscular
- ☐ Tengo problemas de memoria
- ☐ Varias personas cercanas han fallecido
- ☐ Tengo menos energía, me canso más a menudo
- ☐ Mi vista ha empeorado
- ☐ Otro: ____

8. ¿Se siente físicamente débil?

- ☐ Sí, totalmente
- ☐ Bastante
- ☐ Un poco
- ☐ Nada en absoluto

9. Actualmente, ¿sufre limitación en:

	Mucho	Bastante	Un poco	Nada en absoluto
El tiempo de paseo?	☐	☐	☐	☐
El número de veces que va andando a los sitios?	☐	☐	☐	☐
Las distancias de los paseos?	☐	☐	☐	☐
La velocidad de su marcha?	☐	☐	☐	☐
La longitud de sus pasos?	☐	☐	☐	☐

10. Cuando anda...

	Frecuentemente	De vez en cuando	Casi nunca	Nunca	Ya no ando
¿Siente una fatiga importante?	☐	☐	☐	☐	☐
¿Necesita sentarse regularmente para recuperarse?	☐	☐	☐	☐	☐
¿Tiene dificultades para cruzar una carretera o una calle suficientemente rápido?	☐	☐	☐	☐	☐
¿Tiene dificultades en terrenos irregulares?	☐	☐	☐	☐	☐

Copyright© - January 2015 - All right Reserved

11. ¿Tiene problemas de equilibrio?

- ☐ Frecuentemente
- ☐ De vez en cuando
- ☐ Casi nunca
- ☐ Nunca

12. ¿Se cae?

- ☐ Frecuentemente
- ☐ De vez en cuando
- ☐ Casi nunca
- ☐ Nunca

13. ¿Cree usted que su aspecto físico ha cambiado?

- ☐ Sí, totalmente
- ☐ Sí, bastante
- ☐ Sí, un poco
- ☐ No, en absoluto

14. Si la respuesta es sí, ¿de qué manera?

(Puede elegir varias respuestas)

- ☐ Cambios en el peso (aumento o pérdida de peso)
- ☐ Aparición de arrugas
- ☐ Disminución de la talla (altura)
- ☐ Pérdida de masa muscular
- ☐ Pérdida de pelo
- ☐ Aparición de canas/pelo gris
- ☐ Otro:

15. Si la respuesta es sí, ¿se siente inquieto por este cambio?

- ☐ Mucho
- ☐ Bastante
- ☐ Un poco
- ☐ Nada

Copyright© - January 2015 - All right Reserved

16. ¿Tiene la sensación de ser frágil?

- ☐ Sí, totalmente
- ☐ Un poco
- ☐ Nada en absoluto

17. Actualmente, ¿tiene dificultades para realizar estas actividades de la vida cotidiana?

	Totalmente incapaz	Mucha dificultad	Poca dificultad	Sin dificultad	Nunca realizo esta actividad
Subir un tramo de escaleras	☐	☐	☐	☐	☐
Subir varios tramos de escaleras	☐	☐	☐	☐	☐
Subir uno o varios escalones sin barandilla	☐	☐	☐	☐	☐
Ponerse de cuclillas o arrodillarse	☐	☐	☐	☐	☐
Agacharse o inclinarse para recoger un objeto del suelo	☐	☐	☐	☐	☐
Levantarse del suelo sin apoyo	☐	☐	☐	☐	☐
Levantarse de un sillón bajo o una silla sin reposabrazos	☐	☐	☐	☐	☐
Pasar, de forma general, de estar sentado a ponerse de pie	☐	☐	☐	☐	☐
Cargar objetos pesados (bolsa de la compra grande, cacerola llena de agua, etc…)	☐	☐	☐	☐	☐
Abrir una botella o un tarro	☐	☐	☐	☐	☐
Utilizar el transporte público	☐	☐	☐	☐	☐
Subir o bajar de un coche	☐	☐	☐	☐	☐
Hacer la compra	☐	☐	☐	☐	☐
Hacer las tareas del hogar, como hacer la cama, pasar el aspirador, planchar, lavar los platos, etc…	☐	☐	☐	☐	☐

Copyright© - January 2015 - All right Reserved

18. ¿La debilidad muscular limita sus movimientos?

- ☐ Sí, mucho
- ☐ Sí, bastante
- ☐ Sí, un poco
- ☐ No, nada

19. Si la respuesta es sí, ¿por qué razones? (Puede elegir varias respuestas)

- ☐ Por miedo de hacerse daño
- ☐ Por miedo a no poder hacerlo
- ☐ Por miedo a estar cansado después de estas actividades
- ☐ Por miedo a caerse
- ☐ Otros:

20. ¿Su debilidad muscular le impide llevar una vida sexual satisfactoria?

- ☐ Ya no tengo la ocasión de tener vida sexual
- ☐ Sí, totalmente
- ☐ Bastante
- ☐ Un poco
- ☐ Nada en absoluto

21. ¿Ha modificado la práctica de actividad física/deportiva?

- ☐ Ha aumentado
- ☐ Ha disminuido
- ☐ No se ha modificado
- ☐ Nunca he practicado actividad física o deportiva

22. ¿Ha modificado la práctica de actividades de ocio (salir a comer, jardinería, bricolaje, caza/pesca, clubs de jubilados, cartas, pasear, etc.)?

- ☐ Ha aumentado
- ☐ Ha disminuido
- ☐ No se ha modificado
- ☐ Nunca he practicado actividades de ocio

Copyright© - January 2015 - All right Reserved
